# LINE-1 retrotransposons and *let-7* miRNA: partners in the pathogenesis of cancer?

**DOI:** 10.3389/fgene.2014.00338

**Published:** 2014-10-07

**Authors:** Stephen Ohms, Sung-Hun Lee, Danny Rangasamy

**Affiliations:** ^1^Department of Molecular Bioscience, John Curtin School of Medical Research, The Australian National UniversityCanberra, ACT, Australia; ^2^Molecular and Cellular Oncology, University of Texas MD Anderson Cancer CenterHouston, TX, USA

**Keywords:** LINE-1, retrotransposon, *let-7* microRNA, long non-coding RNA, cancer, gene modulation

## Abstract

Long interspersed nuclear element-1 (LINE-1 or L1) retrotransposons are insertional mutagens capable of altering the genomic landscape in many ways. Activation of the normally silent LINE-1 retrotransposon is associated with a high level of cancer-associated DNA damage and genomic instability. Studies of LINE-1 have so far focused mainly on changes in gene expression, and our knowledge of its impact on functional non-coding RNAs is in its infancy. However, current evidence suggests that a significant number of human miRNAs originate from retrotransposon sequences. Furthermore, LINE-1 is generally not expressed in normal tissues while its expression is widespread in epithelial cancers. Based on our recent studies, we demonstrate a functional link between aberrant LINE-1 expression and deregulation of *let-7* miRNA expression. Since the expression of *let-7* is modulated by LINE-1 activity, we discuss possible mechanisms for this effect and how the silencing of LINE-1 activation could provide new therapeutic options for cancer treatment. Based on the deep sequencing of small RNAs in parallel with gene expression profiling in breast cancer cells, we have identified potential pathways linking L1 activity to *let-7* processing and maturation and ultimately to the control of stemness in human cancer cells.

## INTRODUCTION

Retrotransposons, a family of mobile genetic element, are the most common repetitive elements in the human genome. Of these, the long interspersed nuclear element-1 (LINE-1 or L1) and the Alu elements are the most prolific classes of retrotransposon, comprising 28% of the human genomic sequence. L1 is an insertional mutagen capable of copying itself and reinserting into the genome at multiple sites and is thereby capable of wreaking mutational havoc on the genome. The activation of L1 retrotransposons and ensuing L1 retrotransposition is also associated with a high frequency of DNA breaks and genomic instability (Symer et al., 2002) and several studies have shown that there is a direct association between the severity of cancer-associated DNA damage and the activation of L1 expression (Belgnaoui et al., 2006; Wallace et al., 2010). L1 also accelerates the mobilization of Alu elements, certain mRNAs and non-coding RNAs to new sites in the genome (Esnault et al., 2000; Garcia-Perez et al., 2007), further altering cellular function in many ways. Because of these potentially harmful impacts on genomic integrity, normal adult cells have developed a variety of defense mechanisms, including epigenetic silencing, to prevent the expression of L1 elements (Chen et al., 2012b; Rangasamy, 2013). Related to this, studies have shown that hypomethylation of L1 promoters is associated with activation of L1 expression in many types of cancer (Cruickshanks and Tufarelli, 2009). When L1 elements become active, they can rapidly increase in copy number by a “copy-and-paste” mechanism and become a source of genetic mutations. For example, two recent studies have unveiled several tumor-specific *de novo* L1 mutations in lung and liver cancers using the transposable element analyzer (TEA) repeat analysis pipeline and genome-wide mapping (Iskow et al., 2010; Lee et al., 2012).

A recent survey of whole genome sequences from a variety of tumors included in the Cancer Genome Atlas (TCGA) project also identified a number of L1-mediated insertional mutations in colon, prostatic, colorectal, and ovarian cancers, suggesting that L1-induced mutations are common in cancer cells and tissues (Shukla et al., 2013). Despite these findings, questions remain concerning whether the activation of L1 elements is causative of cancer or merely occurs as an epiphenomenon due to the unstable genomic state of cells. Although a clear connection has been established between L1-induced mutations and altered expression of affected genes, it is unclear if these represent cell-type-specific mutations or are sufficiently prevalent to contribute to cancer pathology in general. The activation of L1 retrotransposons occurs mostly in cancers of epithelial origin. In recent studies, we and others have shown that L1 expression occurs in almost all the aggressive forms of human breast cancer characterized by high rates of lymph node metastasis, including estrogen-negative (ER-) tumors, which are characterized by frequent distant metastasis and intrinsic resistance to hormone therapy (Harris et al., 2010; Chen et al., 2012a). In support of these findings, another study has shown that breast carcinomas release retroviral-like particles into the extracellular space that contain high levels of L1-encoded mRNA (Golan et al., 2008). Furthermore, the level of L1 elements is high in the plasma of patients with breast cancer, melanoma, and lymphoma (Balaj et al., 2011), suggesting a link between L1 activity and the recurrent forms of metastasis. Although the contribution of L1 activity to initiating the expression of certain protein-coding oncogenes such as *c-MET*, typically via alternative promoters, has been recognized (Cruickshanks and Tufarelli, 2009; Wolff et al., 2010), little is known about the regulatory role of L1 elements (if any) for non-coding RNA genes. A recent transcriptome study reports that an L1 transcript driven by a viral HBV promoter, referred to as HBx-LINE-1, does not encode a protein but produces a long non-coding RNA (lncRNA) which induces the β-catenin signaling pathway and facilitates the acquisition of a mesenchymal phenotype and metastatic potential (Lau et al., 2014). Strikingly, HBx-LINE-1 expression has been found to occur in ∼25% of hepatocellular carcinomas examined, and correlates with reduced patient survival. Although this finding suggests a role for L1 elements in the development of liver cancer, it is not clear whether a similar pattern of L1-driven lncRNA expression exists in other types of cancer.

## RETROTRANSPOSONS AS THE SOURCE OF NON-CODING RNA

Growing evidence suggests a close association between the presence of retrotransposons in the intergenic regions of the human genome and sources of non-coding RNAs, including miRNAs and lncRNAs. Notably, ∼30% of human lncRNAs originate from retrotransposons, in both sense and antisense orientations. In addition, ∼80% of lncRNAs contain retrotransposon-derived sequences embedded within or nearby their transcription start sites, in which the retrotransposon sequences contribute signals for lncRNA expression, splicing and processing (Kapusta et al., 2013). Genome-wide analyses also reveal that lncRNAs are highly enriched for LTR and HERV elements but are depleted of L1 and Alu elements. Despite the low content of L1-derived sequences, a recent study reported that a point mutation in an L1-containing lncRNA sequence, which is located within an intron of *SLC7A2*, leads to a defect in the expression of the lncRNA and results in a lethal encephalopathy phenotype (Cartault et al., 2012). The presence of this L1 sequence is predicted to contribute to the proper folding of the lncRNA, which is important for its function in the brain.

Alu elements do not encode functional proteins for their mobilization. Instead, Alu rely on the functioning of the L1 machinery. In fact, L1 elements are frequently found overlapping Alu sequences at multiple locations in the genome and in particular, in lncRNA sequences. Several recent studies suggest that Alu elements present in lncRNAs can contribute to the regulatory role of these lncRNAs. One such Alu-mediated lncRNA is *APTR*, which represses *p21* expression by recruiting polycomb repressive proteins to the p21 promoter. The presence of Alu is crucial to the localization of *APTR* to the p21 promoter and thus to regulation of cell growth and proliferation (Negishi et al., 2014). Interestingly, this lncRNA also contains an L1 sequence overlapping with closely spaced pairs of inverted Alu elements. Whether the presence of the L1 sequence has any effects on the functions of the lncRNA remains to be elucidated. The function of Alu elements is also linked to the expression of many disease-related lncRNAs. As a key regulatory element, Alu mediates the expression of an lncRNA, referred to as *ANRIL* (antisense non-coding RNA in the INK4 locus), which binds to polycomb group proteins and interacts with multiple target gene promoters during the process of atherosclerosis (Holdt et al., 2013). The presence of Alu in the lncRNA not only increases the expression of *ANRIL* transcripts but also marks the promoters of target genes for epigenetic silencing. Strikingly, deletion or mutation of the Alu sequence in *ANRIL* normalizes *ANRIL*-regulated gene networks and cellular functions. These findings highlight a new role for retrotransposons in epigenetic *trans*-regulation of gene networks, which might be relevant to other lncRNAs as well.

Another important layer of genetic control that shapes cellular functioning is the expression of microRNAs. Computational studies reveal that miRNA target sites in the 3′-UTRs of genes can be formed from embedded retrotransposon sequences, and also that many miRNAs were initially formed from retrotransposon sequences (Roberts et al., 2014). Most miRNAs are transcribed as long primary transcripts (pri-miRNAs) and processed by Drosha/Dicer to mature miRNAs with lengths of 20–22-nt. miRNAs regulate gene expression post-transcriptionally by binding to one or more mRNAs, ultimately leading to the translational inhibition or degradation of the target genes. Differential expression of miRNAs is observed in many types of cancer with some of these miRNAs playing crucial roles in cancer onset and progression. There is also growing evidence that the seed sequences of miRNAs are derived from retrotransposons (Borchert et al., 2011). For instance, the miRNA-28 family originates from LINE L2B elements. Several computational analyses have reported that some miRNAs share significant sequence homology to retrotransposons (Filshtein et al., 2012). In addition, a substantial number of miRNAs contain hairpin sequences that are related to retrotransposons. In further support of these findings, another recent study has shown that several human miRNAs and miRNA target sites in the 3′-UTRs of genes are, in fact, derived from L1, Alu, and MIR elements (Spengler et al., 2014). Notably, ∼85% of miRNA target sites overlap L1 and Alu elements, indicating that a strong relationship exists between miRNA functionality and the activity of retrotransposons.

**FIGURE 1:**
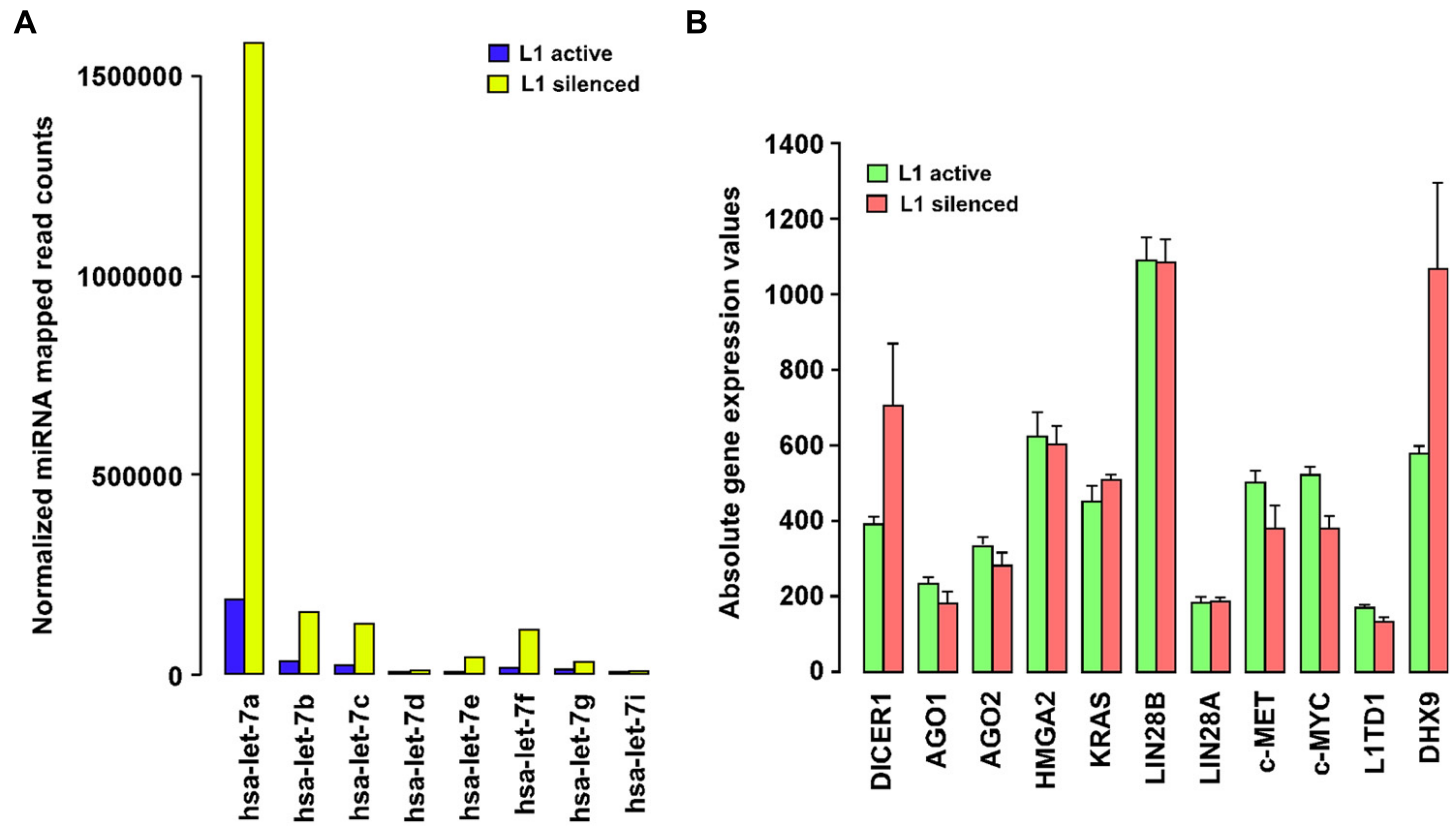
**(A)** Long interspersed nuclear element-1 (L1) silencing modulates the expression of the *let-7* family of miRNAs. Barplot showing DESeq-normalized absolute read counts for *let-7* family miRNAs from L1-silenced small RNA deep sequencing experiment in T47D breast cancer cells. **(B)** Expression profiling of *let-7a*-target gene expression in T47D breast cancer cells before and after silencing L1. Barplot showing normalized absolute gene expression values for selected genes. Error bars show mean ± SEM (*n* = 3 replicates for each experimental group).

Our group has recently shown that L1 elements are not expressed in normal differentiated cells, but that their expression is widespread in all the types of breast tumors and breast cancer cells examined so far, and correlates with poorer patient survival (Chen et al., 2012a). Supplementary Table 1 summarizes the expression of L1 elements determined by different investigators in a variety of cancer cells, tumor tissues, and animal-model studies. To elucidate the molecular functions of L1 elements other than those resulting in insertional mutations, we silenced the expression of endogenous L1 elements in T47D breast cancer cells using an L1-specific endo-siRNA that can specifically silence L1 expression through increased DNA methylation of L1 promoters (Chen et al., 2012b). A genome-wide analysis of miRNA expression using high-throughput deep sequencing showed strong global upregulation of miRNA expression and very marked changes in a number of specific miRNAs secondary to L1 silencing in this cancer cell line (Ohms and Rangasamy, 2014). To our surprise, most of the changes in miRNA expression occur mainly in the *let-7* family of miRNAs (**Figure 1A**). In particular, *let-7a* miRNA was strongly upregulated from 149,428 normalized mapped reads to 1,855,633 reads in L1 silenced cells, accounting for 40% of the increase in the total normalized read counts in the L1-silenced cells compared to cancer cells in which L1 remained active. This massive increase in *let-7a* expression is intriguingly similar to the differential expression of *let-7a* seen in normal cells and a variety of cancer cells in which the expression of *let-7* is repressed (Boyerinas et al., 2010).

*Let-7a* has a highly conserved sequence across organisms from *Caenorhabditis elegans* to humans. It regulates the expression of a range of genes through its 5′ seed sequence (5′-UGAGGUA-3′), which binds to corresponding sequences located in the 3′ UTRs of genes. *Let-7a* is also known to target many oncogenes including *c-Myc, HMGA2*, and *Lin28,* and its expression is a hallmark of cell differentiation. Notably, the loss of *let-7a* expression is often considered to have prognostic value since it indicates poor survival in many cancers. Studies performed in lung and renal cell carcinoma reveal that the overexpression of *let-7a* inhibits *in vitro* cancer cell proliferation and *in vivo* tumor regeneration by reducing the expression of *c-Myc* and c-Myc targeted genes (Liu et al., 2012). Another major target of *let-7a* is *c-MET*, which is one of the key genes activated by L1 expression in cancer cells (Wolff et al., 2010). Abnormal expression of *c-MET* induces multiple signal transduction pathways involved in cancer growth and metastasis including the RAS, PI3K, STAT3, and β-catenin pathways. For these reasons, there is growing interest in the therapeutic use of *let-7a* itself, or pharmacological modulators of *let-7a* to treat human cancers in clinical applications.

Expression of *let-7a* is subjected to complex regulation involving positive (p68/p72 helicases) and negative factors (c-Myc, Lin28, hnRNPA1). The p68/p72 RNA helicases, as components of the Drosha microprocessor complex, stimulate the processing of pri-*let-7* miRNAs into mature RNAs by Dicer-mediated processing. The mature *let-7a* also binds to a complementary region in the pri-*let-7* miRNA, recruiting Argonaute and promoting its own downregulation (Zisoulis et al., 2012). Moreover, the expression of *let-7a* is also controlled by c-Myc binding to the *let-7* promoters which decreases its expression. c-Myc also activates *Lin28* expression by binding to the Lin28 promoters and Lin28, in turn, binds selectively to pri-*let-7* miRNAs and blocks Dicer processing of pri-*let-7* miRNAs into mature *let-7a* (Chang et al., 2009). By repressing *let-7a*, *Lin28* often acts as an oncogene in cancer cells (Viswanathan et al., 2009). Strikingly, Lin28 is itself targeted by *let-7a* thus affecting the functioning of Lin28 in a feedback circuit. Currently, however, few studies have addressed the functional role of L1 in the expression of the *let-7* miRNA family. Thus, to clarify the role of L1, we carried out profiling of *let-7a*-target gene expression, in breast cancer cells before and after silencing L1. This study revealed that L1 silencing reduces the expression of some *let-7a*-targeted genes including c-Myc and c-MET, although only to a modest degree (**Figure 1B**). In another study, inhibition of L1 activity by antiretroviral drugs was shown to reduce c-Myc expression in cancer cells (Sciamanna et al., 2005), which may partially explain the ability of L1-silencing to activate *let-7a*. Importantly, our gene expression profiling shows that Dicer is also significantly upregulated in L1 silenced cells, which supports a recent report of Dicer inhibiting L1 activation in human cells (Heras et al., 2013). What is less clear from these studies is how L1 silencing leads to increased expression of *let-7a* miRNA in cancer cells. Thus, the link between the *let-7a* miRNAs and the expression of L1 elements in cancer cells requires further investigation.

## DOES L1 EXPRESSION INFLUENCE miRNA EXPRESSION?

**FIGURE 2:**
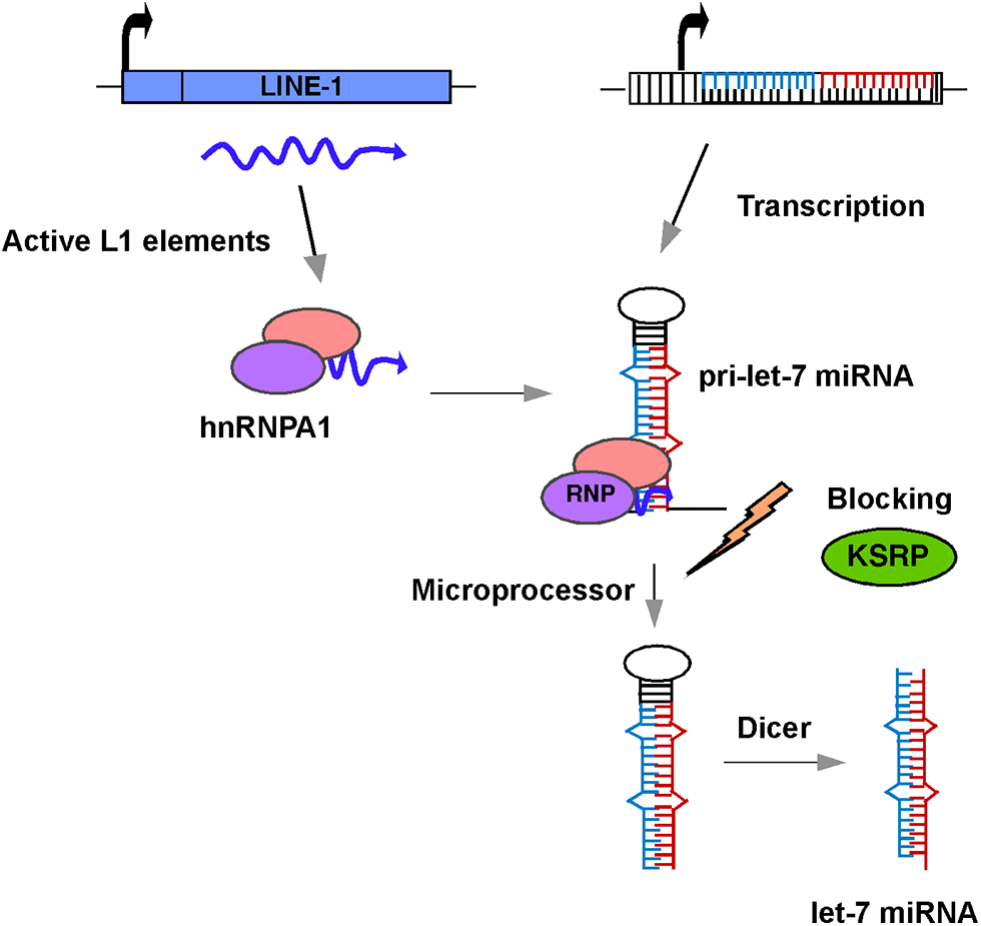
**Schematic diagram of a model showing the role of L1 in *let-7* miRNA processing and maturation.** L1 transcripts in human cancer cells interact with hnRNPA1 to form an L1-hnRNPA1 complex. This complex binds to pre-*let-7* miRNA and prevents recruitment of KSRP regulatory protein thus blocking the action of the microprocessor and preventing the formation of mature *let-7* miRNAs.

There is little or no direct evidence for a reciprocal relationship between the silencing of L1 and *let-7a* expression in the literature. Also, little is known about the relationship between the expression of L1 elements and other miRNAs. So, how might L1 influence the expression of the *let-7a* miRNA? One possible mechanism is that retrotransposon sequences located in the promoter regions of *let-7* miRNAs might act as functional domains for their regulation. Sequence analysis with the RepeatMasker database reveals the presence of retrotransposon fragments scattered throughout the promoter regions of the *let-7* miRNAs, including L1, Alu, and MIR elements. Given that the methylation status of the *let-7* promoters does not appear to play a significant role in the expression of *let-7* miRNAs (Lu et al., 2007), it is conceivable that these inserted retrotransposon transcripts interact with a variety of host proteins, including RNA binding proteins, chromatin modifiers, and regulators of transcription/translation to form an L1 ribonucleoprotein (RNP) complex. Notably, L1 and Alu sequences have recently been shown to confer binding sites for several chromatin regulatory complexes involving lncRNA expression (Blackwell et al., 2012; Goodier et al., 2013). Regardless of whether the inserted retrotransposons within the promoter regions of *let-7* miRNAs are active or not, there are at least 100 copies of highly active L1 elements present in human cells (Brouha et al., 2003). Several lines of evidence indicate that transcripts from these L1 elements are associated with hnRNPA1, which is an abundant RNA binding protein involved in splicing, processing, and export of several pre-mRNAs to the cytoplasm (Goodier et al., 2007; Sokolowski et al., 2013). Importantly, hnRNPA1 also binds to pri-*let-7a* miRNAs and acts as a repressor of *let-7* biogenesis by antagonizing the docking of KSRP (KH-type splicing regulatory protein), which is a component of Drosha/Dicer complexes and is known to positively regulate processing of miRNAs (Michlewski and Caceres, 2010; **Figure 2**). Strikingly, except for pre-*let-7i*, all the members of the *let-7* family interact with hnRNPA1. Thus, there is evidence of a relationship, at least in the case of *let-7a* miRNAs, between the L1 activity and miRNA expression.

An alternative but not mutually exclusive possibility is that L1 silencing may activate miRNA-inducing proteins such as transcription factors and RNA helicases that activate *let-7* biogenesis. Intriguingly, our gene expression microarray profiling of L1-silenced cells showed significantly upregulated expression of the DEAH box-containing DHX9 protein, which shares high sequence similarity to the *Drosophila* maleless protein (MLE), a protein that regulates dosage compensation (Kuroda et al., 1991). DHX9 is an RNA helicase (also known as RNA helicase A, RHA, or NDHII) involved in the RNA-induced silencing complex (RISC) assembly process (Fu and Yuan, 2013) as an RISC-loading factor and this function is mediated by its dsRNA-binding domains. Recently, DHX9 has been shown to interact with Dicer, AGO2, and TRBP2 in miRNA loading and depletion of DHX9 leads to reduced miRNA processing and RISC assembly. Notably, DHX9 also interacts with the L1 RNP complex, along with other RNA helicases, including MOV10, that can potentially impede RISC function (Goodier et al., 2012). Strikingly, a recent study proposed that Lin28 could antagonize the production of *let-7a* miRNAs by recruiting a DHX9-like RNA helicase to promote its own translation (Kallen et al., 2012). Together, these observations suggest that induction of DHX9 by L1 silencing (either directly or through interaction with other proteins) can activate the expression of *let-7a* miRNAs. However, further research is needed to evaluate the precise function of L1 in the activation of the *let-7* miRNAs. Given that *let-7* is often viewed as a tumor suppressor miRNA, a strategy in which L1 activity is selectively inhibited pharmacologically could provide new therapeutic options for human cancer treatment.

In summary, this study has explored the relationship between the expression of L1 elements and cancer onset and progression. In this study we have shown, first, that L1 activity is widespread in epithelial cancers. Second, the expression of non-coding RNAs including miRNAs and lncRNAs is closely associated with L1 activity. Third, we have demonstrated the interplay between the aberrant expression of L1 elements and miRNAs, and in particular, the tumor suppressor miRNA *let-7a*. As we propose above, there is a clear link between *let-7a* expression and the silencing of L1 elements. Questions remain, however, as to how L1 elements, either directly or in combination with other host proteins contribute to the loss of *let-7* expression in the various types of cancer. Further studies are required to thoroughly test the mechanisms proposed above by which L1 might affect miRNA expression. Regardless of the answers to these questions, the available data suggest that silencing of L1 expression holds great therapeutic potential for the treatment of various epithelial cancers.

## Conflict of Interest Statement

The authors declare that the research was conducted in the absence of any commercial or financial relationships that could be construed as a potential conflict of interest.
