# Supplementary material for: LINE-1 retrotransposons and let-7 miRNA: partners in the pathogenesis of cancer?
Source: Front Genet. 2014 Oct 7;5:338. doi: 10.3389/fgene.2014.00338 (PMC4188135; doi:10.3389/fgene.2014.00338)
Supplement: Supplementary file 1 [file Table_1.DOC]

| **Cancer type** | **Experimental Technique** | **Reference** |
| --- | --- | --- |
| Breast cancer cells  Breast tumors | Western blotting  Immunostaining | Chen et al. 2012a |
| Prostate cancer cells  Melanoma cells | Immunofluorescence  Mouse xenograft tumor models | Sciamanna et al. 2005 |
| Lung and brain tumors | Deep sequencing and analysis by TEA (Transposable Element Analyzer) pipeline | Iskow et al. 2010 |
| Prostate, ovarian, colorectal cancers | Whole-genome sequencing and TEA analysis | Lee et al. 2012 |
| Breast cancer, melanoma, lymphoma | Analysis of microvesicles and  Mouse xenograft tumor models | Balaj et al. 2011 |

**Supplementary Table 1**: Expression of L1 elements in human cancer
